# Evaluation of Bone Healing After Enucleation of Jaw Cysts Using Region of Interest (ROI)-Based Gray Value Analysis on Panoramic Radiographs: A Retrospective Study

**DOI:** 10.7759/cureus.108377

**Published:** 2026-05-06

**Authors:** Halil Ibrahim Durmus, Ümit Solmaz, Kagan Fatih Kusan, Tevfik Kizilseki, Nuran Dag

**Affiliations:** 1 Department of Oral and Maxillofacial Surgery, Faculty of Dentistry, Adıyaman University, Adıyaman, TUR

**Keywords:** cystic jaw lesion, enucleation, gray value analysis, imagej, panoramic radiography

## Abstract

Introduction: The aim of this study is to evaluate bone healing after enucleation of cystic jaw lesions using region of interest (ROI)-based gray value analysis on panoramic radiographs. Additionally, this study aims to quantitatively assess early postoperative changes in the lesion area and to determine whether grayscale values approach those of the contralateral healthy bone. Furthermore, it seeks to investigate the applicability of panoramic radiography as a reliable and accessible tool for objective radiographic follow-up in routine clinical practice. Therefore, grayscale analysis provides an indirect, quantitative surrogate of bone healing based on relative pixel intensity changes, rather than a direct histological or volumetric assessment of bone healing.

Methodology: In this retrospective study, 15 patients were selected from approximately 200 patients who presented to our clinic between June 1, 2025, and February 15, 2026, with cystic lesions ≥1.5 cm treated solely by enucleation. Postoperative panoramic radiographs were obtained three to six months after surgery. All images were acquired using the same digital panoramic system under fixed parameters (65 kVp, 5 mA, 17 s). Diagnosis was confirmed histopathologically, and all cases were radicular or dentigerous cysts. Image analysis was performed using ImageJ software (v1.54k; National Institutes of Health, Bethesda, MD). Standardized ROIs (60 × 60 pixels) were defined in the lesion area and the contralateral control area on pre- and postoperative radiographs. Gray values were measured twice and averaged.

Results: The mean grayscale value of the lesion area increased from 77.54 ± 31.19 preoperatively to 94.57 ± 34.05 postoperatively, representing an approximate 22% increase (*P *< 0.001). In addition, postoperative grayscale values in the lesion region approached those of the contralateral control areas. The observed change demonstrated a large effect size, indicating that the radiographic improvement was not only statistically significant but also clinically meaningful.

Conclusions: ROI-based grayscale analysis is a practical, reproducible, and cost-effective method for assessing postoperative radiographic changes related to bone healing after enucleation of cystic jaw lesions. This analysis, performed via panoramic radiographs, provides objective and reliable data for clinical follow-up. Furthermore, this approach may serve as a cost-effective and accessible alternative for quantitative monitoring of early bone healing in routine clinical practice, especially when advanced imaging modalities are not readily available.

## Introduction

Cystic jaw lesions are pathological formations that can be of odontogenic or non-odontogenic origin, can lead to destruction of the jaw bones, and are often clinically asymptomatic [1,2]. The most common odontogenic cysts include radicular cysts and dentigerous cysts, and these lesions are usually detected incidentally during routine radiographic examinations [3,4]. The most common approach in the treatment of cystic lesions is surgical enucleation, and the healing process of the bone defect created after this procedure is evaluated clinically and radiographically [5,6].

Accurate evaluation of postoperative bone healing is of critical importance in determining treatment success, guiding clinical decision-making, and planning further interventions when necessary [5,6]. In routine practice, radiographic follow-up remains the most accessible method for monitoring healing; however, the lack of standardized and objective criteria may lead to variability in interpretation among clinicians [5]. Therefore, there is an increasing need for simple, reproducible, and quantitative methods that can be integrated into daily clinical workflows without requiring additional imaging modalities or increased radiation exposure [6].

Although conventional radiographic methods are widely used in the assessment of bone healing, these methods are mostly based on subjective interpretations [7]. With the development of digital imaging technologies, it has become possible to perform quantitative analysis from radiographic images. In this context, gray value analysis stands out as an objective and reproducible method used in the assessment of bone density and mineralization level [8,9].

Grayscale measurements obtained using open-source image analysis software such as ImageJ (National Institutes of Health, Bethesda, MD) allow for the quantitative assessment of changes in bone tissue [10]. In particular, region of interest (ROI)-based analyses allow for the standardized examination of density changes in a specific anatomical region [11]. However, panoramic radiographs have variations due to magnification, distortion, and patient position, and it is reported that these effects can be minimized with intra-patient comparisons [7].

Within this framework, the aim of this study is to quantitatively evaluate bone healing after enucleation of cystic jaw lesions using ROI-based grayscale analysis on orthopantomograms. Therefore, grayscale analysis provides an indirect, quantitative surrogate of bone healing based on relative pixel intensity changes, rather than a direct histological or volumetric assessment of bone healing [7].

## Materials and methods

Study design and patient selection

Patients with cystic jaw lesions measuring ≥1.5 cm in diameter on orthopantomogram and treated solely by surgical enucleation were included in this study. All included cases had available preoperative and postoperative (3-6 months) orthopantomograms and a definitive histopathological diagnosis of either radicular or dentigerous cysts.

Patients were excluded if radiographic records were incomplete, if alternative surgical techniques such as marsupialization or decompression had been performed, or if systemic diseases affecting bone metabolism were present. In addition, radiographs with low quality or artifacts and cases with postoperative complications that could influence bone healing (e.g., infection) were also excluded.

To minimize the potential effects of systemic conditions on bone metabolism, patients with known metabolic bone diseases (e.g., osteoporosis), uncontrolled diabetes mellitus, or a history of medications affecting bone turnover (such as bisphosphonates or corticosteroids) were excluded from the study. In addition, patients with a history of smoking, alcohol abuse, or any systemic condition that could impair bone healing were also excluded. The inclusion and exclusion criteria were defined to ensure a more homogeneous study population and to reduce potential confounding factors affecting bone healing outcomes.

**Figure 1:**
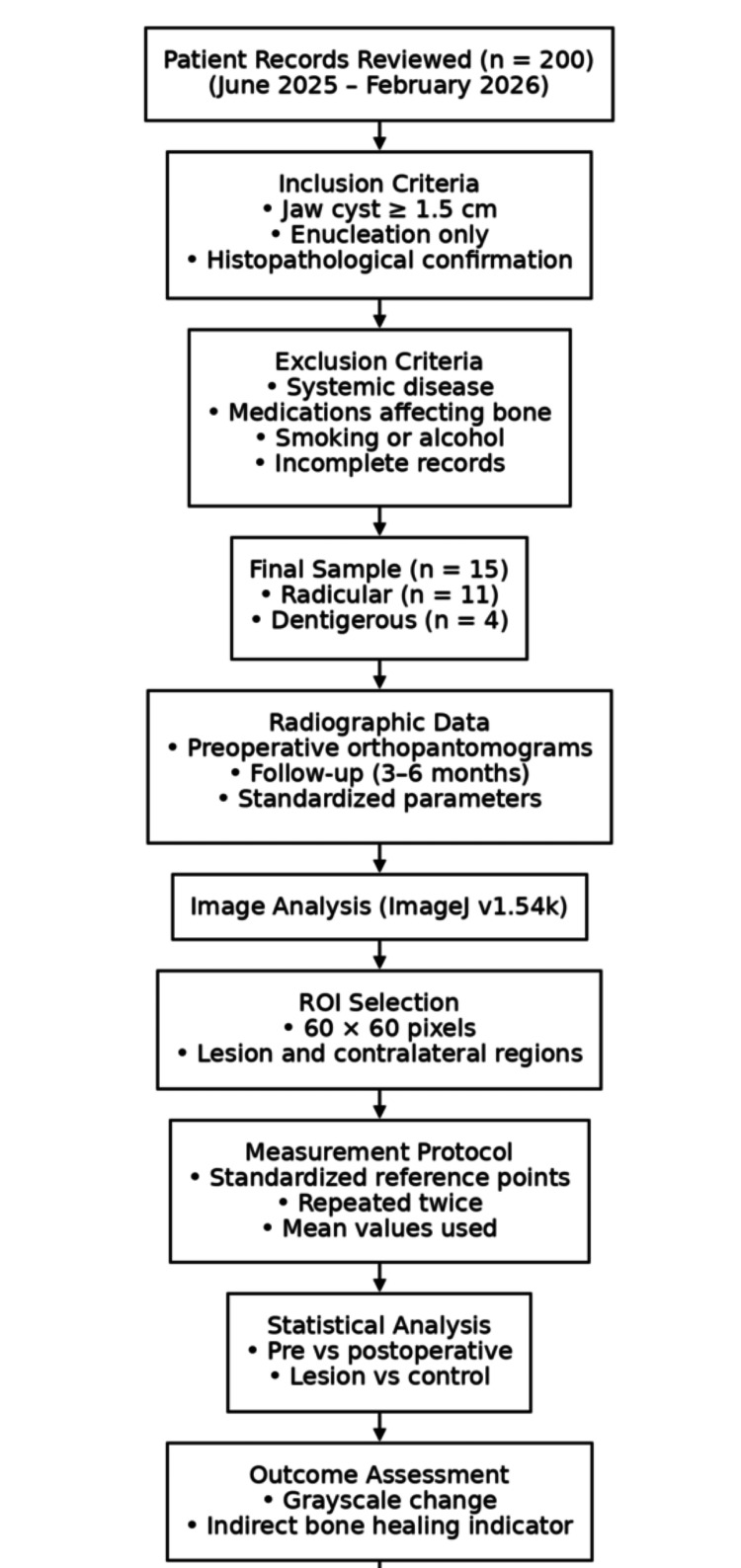
Flowchart of the study methodology. This flowchart summarizes patient selection, inclusion and exclusion criteria, radiographic data acquisition, ROI-based grayscale analysis using ImageJ software, and statistical evaluation of preoperative and postoperative grayscale changes. Image credit: All authors. ROI, region of interest

A flowchart summarizing the study design, patient selection process, radiographic acquisition, and ROI-based grayscale analysis is presented in Figure 1.

Radiographic imaging and image analysis

All orthopantomograms were obtained using the same digital panoramic imaging system under standardized parameters (65 kVp, 5 mA, 17 s) by an experienced technician. Patient positioning was performed according to the manufacturer’s guidelines, and efforts were made to maintain consistent positioning between preoperative and postoperative radiographs. The images were exported in uncompressed TIFF format at a resolution of 300 dpi.

Image analysis was performed using ImageJ software (version 1.54k). Spatial calibration was not applied because grayscale measurements were based on relative pixel intensity values, and ROI were defined in pixels and kept constant across all images to ensure methodological consistency.

For each patient, standardized square ROIs of 60 × 60 pixels were defined in both the lesion area and the contralateral control region on preoperative and postoperative radiographs. ROIs were placed in the most radiolucent area of the lesion, ensuring that the entire ROI was confined within the cystic cavity while avoiding cortical borders and adjacent anatomical structures. In the contralateral region, ROIs of identical size were positioned using consistent anatomical landmarks as reference points to ensure comparability.

To improve reproducibility, ROI placement criteria, size, and anatomical reference points were standardized across all radiographs. Mean grayscale values were calculated for each ROI. All measurements were performed twice by the same observer at a two-week interval, and the average of these measurements was used for statistical analysis.

Statistical analysis

The normality of the obtained data was evaluated using the Shapiro-Wilk test [12]. For data showing a normal distribution, a paired-samples t-test was applied to examine differences between preoperative and postoperative measurements obtained from the same individuals. Grayscale measurements for lesion and control areas were expressed as mean ± standard deviation (mean ± SD). To increase the intra-observer reliability of the measurements, all grayscale assessments were performed twice by the same researcher, and the average of these two measurements was used in the analyses.

Statistical analyses were performed using SPSS (version 25.0, IBM Corp., Armonk, NY) software, and the statistical significance level was set at *P *< 0.05. The obtained data were evaluated comparatively between preoperative and postoperative measurements and values ​​obtained from control areas.

A post hoc power analysis was performed using G*Power software (version 3.1, Heinrich Heine University, Düsseldorf, Germany). Based on a paired-sample t-test design, an effect size (Cohen’s d) of 1.32, a total sample size of 15, and an alpha level of 0.05, the statistical power (1 − β) was calculated as 0.99, indicating a high level of statistical power. This result suggests that the sample size was sufficient to detect the observed effect. Intra-observer reliability was assessed using the intraclass correlation coefficient (ICC). The ICC value was found to be 0.94, indicating excellent reliability [13]. A post hoc power analysis demonstrated that the statistical power of the study was 0.99.

## Results

A total of 15 patients were included in the study. Among the lesions included, 8 (53.3%) were radicular cysts and 7 (46.7%) were dentigerous cysts. Grayscale measurements obtained from preoperative and postoperative panoramic radiographs were compared to evaluate bone healing in the lesion areas. The baseline characteristics of the patients are presented in Table 1.

**Table 1:** Baseline demographic and clinical characteristics of the study population.

Variable	Value
Number of patients (*n*)	15
Mean age (years)	34.9 ± 15.6 (range: 15-67)
Sex (Male/Female)	7/8
Lesion type, *n* (%)	
Radicular cyst	8 (53.3%)
Dentigerous cyst	7 (46.7%)
Lesion size (≥1.5 cm)	15 (100%)
Follow-up period	3-6 months

The mean grayscale value of the lesion area increased from 77.54 ± 31.19 in the preoperative period to 94.57 ± 34.05 in the postoperative period. This increase corresponded to an approximate 22% relative rise in grayscale values and was found to be statistically significant (*P *< 0.001) according to the paired-samples t-test.

The mean difference between preoperative and postoperative grayscale values in the lesion region was 17.03, with a 95% confidence interval (CI) of 9.89-24.15. The calculated Cohen’s d effect size was 1.32, indicating a large effect size.

In contrast, no statistically significant difference was observed in the contralateral control areas. The mean grayscale value of the control region was 117.92 ± 34.69 preoperatively and 119.58 ± 36.23 postoperatively (*P* = 0.47).

These findings indicate that the increase in grayscale values was specific to the lesion region rather than representing a generalized change across the radiograph.

A detailed comparison of preoperative and postoperative grayscale values is presented in Table 2.

**Table 2:** Comparison of preoperative and postoperative grayscale values. Values are presented as mean ± standard deviation (mean ± SD). Statistical analysis was performed using a paired-sample t-test.

Region	Pre-op (Mean ± SD)	Post-op (Mean ± SD)	Mean difference	95% CI	*P*-value	Cohen’s d
Lesion	77.54 ± 31.19	94.57 ± 34.05	17.03	9.89–24.15	<0.001	1.32
Control	117.92 ± 34.69	119.58 ± 36.23	1.66	-	0.47	-

**Figure 2:**
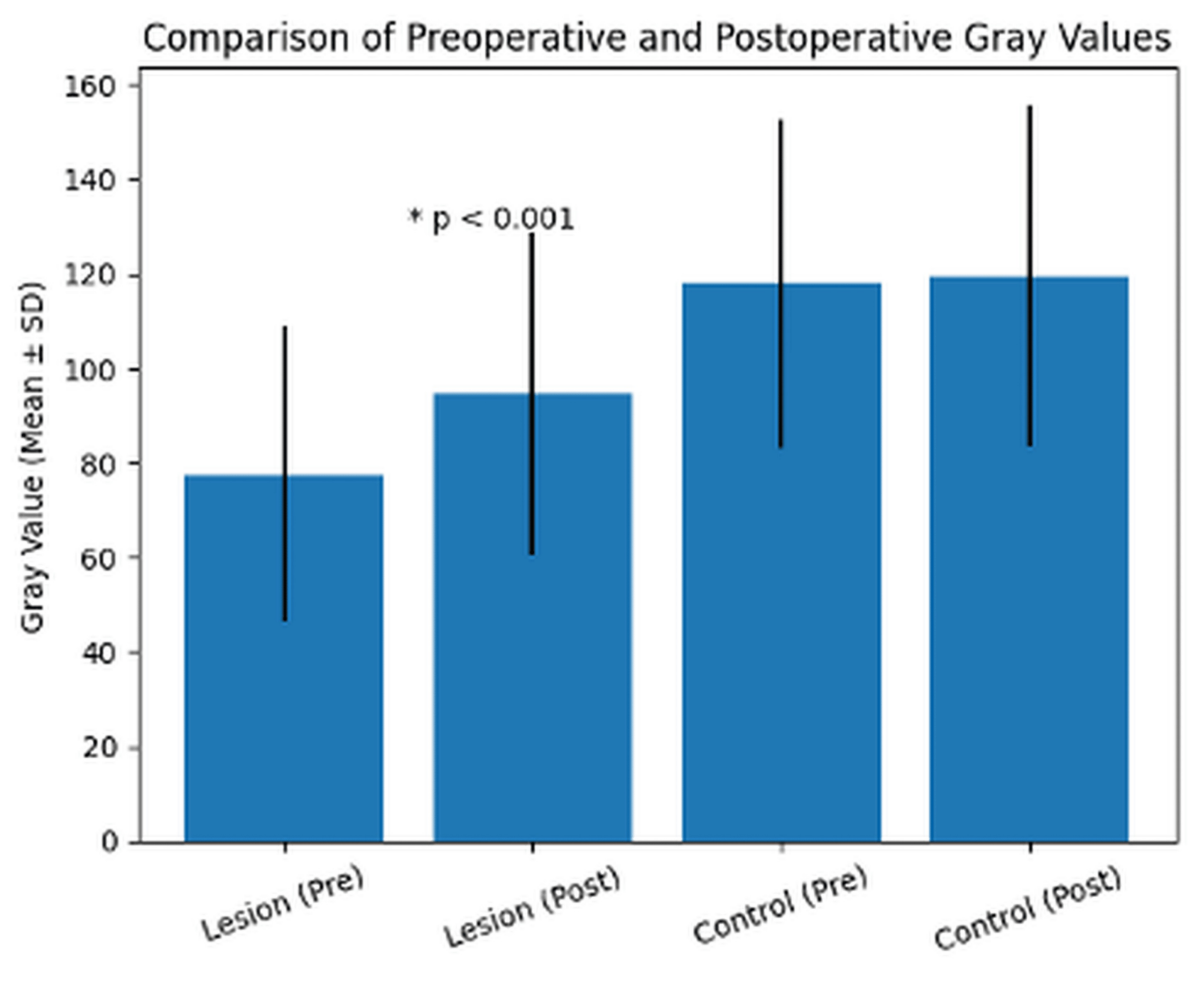
Comparison of preoperative and postoperative grayscale values in lesion and control areas.

Figure 2 illustrates the change in grayscale values between the preoperative and postoperative measurements in both lesion and control regions.

**Figure 3:**
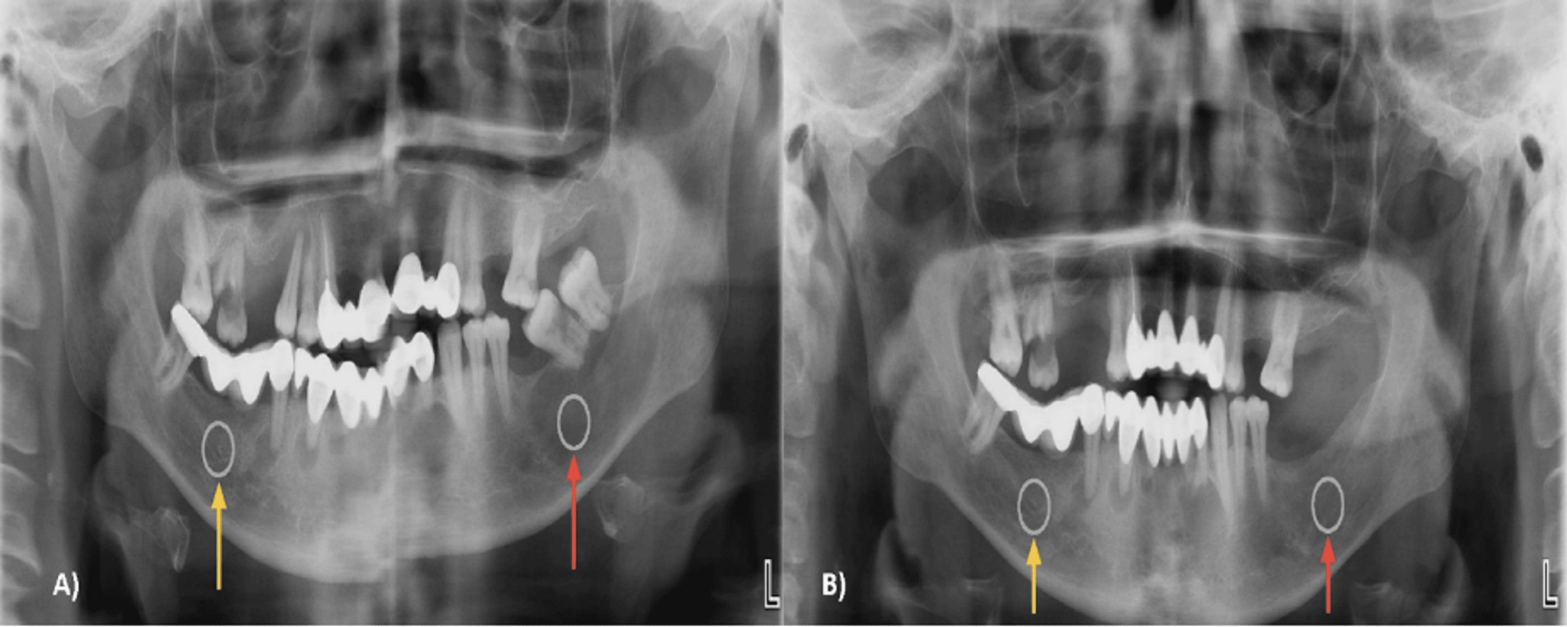
Preoperative (A) and postoperative (B) panoramic radiographs demonstrating standardized ROI placement. Yellow arrows indicate the contralateral control region, while red arrows indicate the lesion area used for grayscale analysis. ROI, region of interest

Preoperative and postoperative panoramic radiographs demonstrating standardized ROI placement in the lesion and contralateral control areas are presented in Figure 3.

## Discussion

In this study, bone healing following enucleation of cystic lesions in the jaws was quantitatively evaluated using ROI-based grayscale analysis on orthopantomograms. The findings revealed a significant increase in grayscale values ​​in the lesion area in the postoperative period, indicating increased bone regeneration in the defect region. However, it should be noted that grayscale values reflect relative changes in pixel intensity and should be interpreted as an indirect indicator of bone healing rather than a direct measure of true bone healing [8,9].

In the literature, it is reported that small cystic lesions can be followed with more conservative approaches, whereas as the size of the lesion increases, findings such as bone expansion, cortical thinning, and involvement of surrounding anatomical structures occur [4,5]. In addition, it has been shown that the treatment approach of large cystic lesions varies depending on the size and often requires surgical intervention, while in some cases, additional treatment methods such as decompression may be planned first [14,15]. These findings indicate that lesion size is a critical factor in treatment planning. Therefore, in our study, clinically significant cases requiring surgical treatment were targeted using a threshold value of ≥1.5 cm [16].

The main reason for choosing ImageJ software is that it is an open-source, reproducible, and reliable platform that allows for quantitative analysis based on pixel density. Previous studies have shown that grayscale analysis in digital radiographs can be used to assess bone density and provides reliable results [9-11]. This allows for more objective results compared to subjective assessments. Similar studies have reported that grayscale analysis and other quantitative image-based methods can be used to monitor bone healing after surgical procedures, supporting the findings of the present study [8,9,17].

To improve measurement consistency, a standardized ROI size was used for all measurements. Previous radiographic studies have reported that maintaining consistent ROI dimensions improves the reliability and reproducibility of grayscale measurements [7-9]. This approach helps ensure that observed variations in grayscale values ​​reflect actual changes in bone density rather than differences related to the measurement area. In addition, ROI placement was carefully standardized by positioning the regions within the most radiolucent area of the lesion and ensuring that the entire ROI remained within the cystic cavity, while corresponding contralateral ROIs were defined using consistent anatomical reference points [8,11].

A follow-up period of three to six months was selected to evaluate early radiographic changes associated with bone healing. Previous studies have shown that trabecular bone formation and mineralization can become radiographically detectable during the early postoperative period following cyst enucleation [11]. In addition, several studies have reported that significant radiographic bone fill and density changes may occur within three to six months after surgery [5,11,15]. These findings support that the postoperative interval used in the present study is sufficient for evaluating early bone healing. In our clinical routine, patients are typically followed up at three or six months after cyst enucleation, depending on clinical indications. Therefore, the inclusion of radiographs obtained at these time points reflects real-world clinical practice rather than a strictly standardized prospective follow-up protocol. Additional radiographic follow-up is not routinely performed in cases where clinical symptoms have resolved, and radiographic bone healing is already evident, in order to avoid unnecessary radiation exposure [5,7].

Histopathological confirmation of the lesions included in the study (radicular and dentigerous cysts) increases diagnostic accuracy and supports the reliability of the findings [1,2]. Moreover, the large effect size observed in this study indicates that the increase in grayscale values is not only statistically significant but also clinically meaningful [18]. Reporting confidence intervals also improves the interpretability of the results by indicating the potential range of the observed effect [19].

One of the most important contributions of this study is demonstrating that bone healing can be quantitatively evaluated using routine imaging such as panoramic radiographs. Orthopantomogram is routinely used in dental practice and provides a practical method for postoperative follow-up compared with more advanced imaging modalities such as cone beam computed tomography (CBCT). This method provides a practical and cost-effective approach for postoperative monitoring and may serve as an accessible alternative to advanced imaging techniques [20-22]. The observed increase in grayscale values suggests that clinically relevant bone regeneration can be detected within the first three to six months after surgery [23].

Despite these findings, several limitations should be considered. Orthopantomograms are two-dimensional images and may be affected by magnification and geometric distortion [24,25]. In addition, grayscale values represent relative pixel density rather than absolute bone mineral density, and variations in patient positioning may influence measurements [26]. Another limitation of this study is the relatively small sample size. Furthermore, the retrospective design and relatively short follow-up period may limit the generalizability of the findings. Although all measurements were performed by a single observer to ensure consistency, inter-observer variability and blinding were not assessed, which may introduce potential bias. Additionally, the lack of external calibration for grayscale values represents another methodological limitation [9,13].

## Conclusions

The findings of this study indicate that postoperative changes in bone density following cyst enucleation can be quantitatively evaluated using ROI-based grayscale analysis on orthopantomograms. However, grayscale values represent relative changes in pixel intensity and should be interpreted as an indirect indicator of bone healing rather than a direct measure of true bone healing.

Within these limitations, this method provides a practical, reproducible, and cost-effective approach for radiographic follow-up in clinical practice. Further studies with larger sample sizes, standardized imaging protocols, and longer follow-up periods are needed to validate and extend these findings.

## References

[1] Soluk-Tekkesin M, Wright JM (2022). The World Health Organization classification of odontogenic lesions: a summary of the changes of the 2022 (5th) edition. Turk Patoloji Derg.

[2] Singh HP, Chahal GK, Sharma G, Gandhi P (2024). A systematic review on odontogenic cysts and tumours. J Oral Maxillofac Pathol.

[3] Johnson NR, Gannon OM, Savage NW, Batstone MD (2014). Frequency of odontogenic cysts and tumors: a systematic review. J Investig Clin Dent.

[4] Daley TD, Wysocki GP (1995). The small dentigerous cyst: a diagnostic dilemma. Oral Surg Oral Med Oral Pathol Oral Radiol Endod.

[5] Stoelinga PJ (2012). The management of aggressive cysts of the jaws. J Maxillofac Oral Surg.

[6] Jeong YH, Kim JS, Lee H, Ahn KM (2025). Comparison of recurrence rates of odontogenic keratocyst and ameloblastoma following surgical excision and peripheral ostectomy in the maxilla. Maxillofac Plast Reconstr Surg.

[7] Boeddinghaus R, Whyte A (2008). Current concepts in maxillofacial imaging. Eur J Radiol.

[8] Maciel ER, Nascimento EH, Gaêta-Araujo H, Pontual ML, Pontual AD, Ramos-Perez FM (2022). Automatic exposure compensation in intraoral digital radiography: effect on the gray values of dental tissues. BMC Med Imaging.

[9] Nomura Y, Watanabe H, Honda E, Kurabayashi T (2010). Reliability of voxel values from cone-beam computed tomography for dental use in evaluating bone mineral density. Clin Oral Implants Res.

[10] Schneider CA, Rasband WS, Eliceiri KW (2012). NIH Image to ImageJ: 25 years of image analysis. Nat Methods.

[11] Southard KA, Southard TE (1994). Detection of simulated osteoporosis in human anterior maxillary alveolar bone with digital subtraction. Oral Surg Oral Med Oral Pathol.

[12] Alonso JC, Montenegro S (2015). Estudio de Monte Carlo para comparar pruebas de normalidad sobre residuos de mínimos cuadrados ordinarios en presencia de procesos autorregresivos de primer orden. Estud Gerenc.

[13] Koo TK, Li MY (2016). A guideline of selecting and reporting intraclass correlation coefficients for reliability research. J Chiropr Med.

[14] Marker P, Brøndum N, Clausen PP, Bastian HL (1996). Treatment of large odontogenic keratocysts by decompression and later cystectomy: a long-term follow-up and a histologic study of 23 cases. Oral Surg Oral Med Oral Pathol Oral Radiol Endod.

[15] Nakamura N, Mitsuyasu T, Mitsuyasu Y, Taketomi T, Higuchi Y, Ohishi M (2002). Marsupialization for odontogenic keratocysts: long-term follow-up analysis of the effects and changes in growth characteristics. Oral Surg Oral Med Oral Pathol Oral Radiol Endod.

[16] Bataineh AB, Al Qudah M (1998). Treatment of mandibular odontogenic keratocysts. Oral Surg Oral Med Oral Pathol Oral Radiol Endod.

[17] Kato CN, Barra SG, Tavares NP, Amaral TM, Brasileiro CB, Mesquita RA, Abreu LG (2020). Use of fractal analysis in dental images: a systematic review. Dentomaxillofac Radiol.

[18] Sullivan GM, Feinn R (2012). Using effect size-or why the P value is not enough. J Grad Med Educ.

[19] Gardner MJ, Altman DG (1986). Confidence intervals rather than P values: estimation rather than hypothesis testing. Br Med J (Clin Res Ed).

[20] Scarfe WC, Li Z, Aboelmaaty W, Scott SA, Farman AG (2012). Maxillofacial cone beam computed tomography: essence, elements and steps to interpretation. Aust Dent J.

[21] Pauwels R, Jacobs R, Singer SR, Mupparapu M (2015). CBCT-based bone quality assessment: are Hounsfield units applicable?. Dentomaxillofac Radiol.

[22] Scarfe WC, Farman AG (2008). What is cone-beam CT and how does it work?. Dent Clin North Am.

[23] de Molon RS, Batitucci RG, Spin-Neto R, Paquier GM, Sakakura CE, Tosoni GM, Scaf G (2013). Comparison of changes in dental and bone radiographic densities in the presence of different soft-tissue simulators using pixel intensity and digital subtraction analyses. Dentomaxillofac Radiol.

[24] Perschbacher S (2012). Interpretation of panoramic radiographs. Aust Dent J.

[25] Devlin H, Yuan J (2013). Object position and image magnification in dental panoramic radiography: a theoretical analysis. Dentomaxillofac Radiol.

[26] Subbulakshmi AC, Mohan N, Thiruneervannan R, Naveen S (2016). Positioning errors in digital panoramic radiographs: a study. J Orofac Sci.

